# Editorial: Antimicrobial resistance and antimicrobial alternatives

**DOI:** 10.3389/fmed.2023.1171283

**Published:** 2023-03-06

**Authors:** Marwan Osman, Imad Al Kassaa, Khaled El Omari, Issmat I. Kassem

**Affiliations:** ^1^Cornell Atkinson Center for Sustainability, Cornell University, Ithaca, NY, United States; ^2^Department of Public and Ecosystem Health, College of Veterinary Medicine, Cornell University, Ithaca, NY, United States; ^3^Biosciences Team, Fonterra Research and Development Center, Palmerston North, New Zealand; ^4^Quality Control Center Laboratories at the Chamber of Commerce, Industry & Agriculture of Tripoli & North Lebanon, Tripoli, Lebanon; ^5^Laboratoire Microbiologie Santé et Environnement (LMSE), Doctoral School of Sciences and Technology, Faculty of Public Health, Lebanese University, Tripoli, Lebanon; ^6^Center for Food Safety and Department of Food Science and Technology, University of Georgia, Griffin, GA, United States

**Keywords:** antimicrobial resistance (AMR), One Health, molecular epidemiology, sustainability, refugee crisis, infection prevention and control (IPC), water sanitation and hygiene (WASH), surveillance

Antimicrobial resistance (AMR) poses a relentlessly growing threat to human and animal health across the globe. The inappropriate use of antimicrobials in clinical and agricultural settings has created suitable conditions for the evolution, emergence, and dissemination of AMR. Overreliance on antimicrobials has resulted in an unprecedented selection pressure that spurred the proliferation of antimicrobial-resistant microorganisms in human, animal, and environmental microbiota (1). These conditions also facilitate the mobilization and horizontal transfer of resistance determinants from commensal bacteria to those that can cause disease; especially with the accumulation of evolutionary events that select for resistance in host populations and environmental ecosystems. Today, AMR is casting an ominous shadow; a critical health challenge with magnitudes dwarfing most life-threatening diseases such as HIV and malaria (2). It has been famously predicted that 10 million people could die annually by 2050 if robust interventions to control AMR were not adopted (2). Recently, a comprehensive assessment showed that AMR directly caused an estimated 1.27 million deaths and was associated with 4.95 million deaths globally (2). Taken together, AMR is a current and serious threat that might cause a global crisis, shifting the balance of the war against infectious diseases away from human and animal welfare. Unifying efforts to tackle the scourge of AMR are urgently required; including robust science-based monitoring of AMR transmission using the One Health ethos and investigating new advances in non-traditional and next-generation antimicrobial products that reduce reliance on conventional antimicrobials.

This Research Topic: “*Antimicrobial resistance and antimicrobial alternatives*” hosts 11 manuscripts, including seven original articles, one systematic review, two narrative reviews, and one perspective article. The manuscripts were contributed by 76 authors from various institutions and research centers. This Research Topic is involved in addressing AMR challenges by assessing trends in antimicrobial use and the development of resistance, especially in developing countries, and exploring the antimicrobial properties of promising non-traditional products. Inconsistent antimicrobial stewardship programs and deficiencies in systemic surveillance of AMR in low- and middle-income countries (LMICs) have promoted the injudicious use of antimicrobials among humans and other animals. Consequently, multidrug-resistant (MDR) microorganisms have been steadily increasing (1). At the community level, Khan et al. showed that storage of antimicrobials is common in households in post-conflict regions in Pakistan, and the communities also suffer from a lack of awareness on antimicrobial use and AMR. At the hospital level, Sirilak et al. suggested the need for reducing the unnecessary use of antimicrobials by adopting the recommendations of the World Health Organization and revising local guidelines on the empirical treatment of postpartum infections in women with episiotomy lesions in Thailand, an upper-middle income country. Sirilak et al. reported no significant differences in postpartum infections between patients that received antimicrobial treatments in comparison to those who did not.

Although the highest toll of AMR burden is thought to affect developing countries (2), it is well-known that drug-resistant microorganisms can easily spill over across geographic borders to affect both developing and developed countries. Unfortunately, limited data have been published on the epidemiology of AMR in LMICs, particularly among vulnerable populations (e.g., refugees) and in non-clinical settings (e.g., community, environment). Moreover, currently available data are usually incomplete and might not provide a comprehensive representation of the AMR burden, because the findings are based on small-scale studies and are temporally and spatially limited due to the lack of resources. Therefore, it was very pertinent that Osman et al. argued that vulnerable populations, specifically refugees and their hosting communities in conflict zones, in the Middle East and beyond are at an elevated risk of life-threatening AMR infections. For example, Syrian refugees in makeshift camps and other disenfranchised populations in Lebanon are susceptible to infectious diseases and antimicrobial-resistant pathogens, which are amplified by COVID-19 and dire social and economic situations. In Lebanon, MDR Gram-negative bacterial infections have been reported in critical patients diagnosed with COVID-19 in clinical settings (Sleiman et al.). Interestingly, the mobile colistin resistance gene (*mcr-1.26*), previously isolated in a pigeon in Lebanon (3), was reported in a Lebanese hospital (Sleiman et al.). The spread of MDR isolates (including carbapenem- and third-generation cephalosporin-resistant strains) is of great concern, especially in LMICs (e.g., Lebanon) and the disenfranchised population that are experiencing compounded public health challenges. The findings corroborated those observed in China, which reported a rapid increase in the fecal carriage rate of extended-spectrum beta-lactamase (ESBL)-producing *Klebsiella pneumoniae* among healthy rural residents (Wang et al.). The most common AMR determinants documented in these residents were *bla*_CTX−*M*−14_, followed by *bla*_CTX−*M*−3_, *bla*_CTX−*M*−15_, *bla*_CTX−*M*−27_, *bla*_CTX−*M*−24_, and *bla*_CTX−*M*−65_ (Wang et al.). To halt the dissemination of AMR in these socioeconomic settings, it is crucial to understand both the intrinsic and extrinsic factors that contribute to the emergence and persistence of resistance. Therefore, there is a paramount need for One Health-based surveillance studies that allow local and international stakeholders to improve AMR stewardship programs and address the calamitous problems that threaten these populations.

Given the rapid spread of resistance, it is imperative to seek novel antimicrobial agents and other therapeutic options to overcome increasing rates of AMR and treat life-threatening MDR infections. Eravacycline, a relatively new fluorocycline antimicrobial with broad-spectrum efficacy against common clinical pathogens, was not inferior to ertapenem and meropenem in adult patients with complicated intra-abdominal infections. Subsequently, Eravacycline might represent an excellent option for the treatment of these infections (Meng et al.). Although there is a pressing demand for new antimicrobials, the use of antimicrobial alternatives (i.e., non-antibiotic antimicrobial therapies) is also sorely needed, especially when noting the decline in the discovery and development of novel antimicrobial agents. It is no secret that pharmaceutical companies are reluctant to develop new drugs due to regulatory, scientific, and financial barriers (4). In this context, current efforts must expand toward promoting next-generation anti-infectives along with reducing the unnecessary use of antimicrobials. Vaccines and alternative therapies (i.e., bacteriophages, antimicrobial peptides, bacteriocins, lysins, CRISPR/Cas9, antibodies, antimicrobial adjuvants, probiotics, and microbiome alterations) must be explored and adopted as first-line options for infection prevention and treatment. For example, Blue light is emerging as a safe microbicidal tool in several clinical and other public health-associated applications, such as food safety, environmental decontamination, and treatment of clinically relevant pathogens (Haridas and Atreya). Furthermore, Gungordu Er et al. highlighted that graphene-based nanomaterials can be potential antiviral candidates for biomedical applications such as antimicrobial personal protective equipment. Future investigations must continue to explore more promising antimicrobial alternatives that can effectively control untreatable complicated infections, perhaps tackling etiologic agents in a variety of settings in the host and beyond. While progress is being made, the current challenge is to implement these alternative therapies in medical practices, prove their efficacy, safety, and affordability, and gradually replace/supplement conventional antimicrobial interventions.

In conclusion, a multi-pronged approach that engages all stakeholders (including scientists, legislators, clinicians, pharmaceutical companies, and the public) is necessary to avoid the tsunami of AMR. Resistance is not only a local challenge, but it also affects everyone across the globe and requires holistic consideration of clinical, agricultural, and environmental practices. Special and real support must be provided to protect the most vulnerable populations in locations where AMR can take hold, amplify, and spread; causing unimaginable suffering. The manuscripts published on this Research Topic highlighted the growing problems of inappropriate use of antimicrobials and the spread of AMR and the need for effective antimicrobial alternatives.

## Author contributions

MO drafted and edited the manuscript. IA, KE, and IK edited the manuscript. All authors approved it for publication.
